# Crystal structure of 4-(tri­methyl­germ­yl)benzoic acid

**DOI:** 10.1107/S2056989015009160

**Published:** 2015-05-23

**Authors:** Lena Knauer, Eva R. Barth, Christopher Golz, Carsten Strohmann

**Affiliations:** a Fakultät für Chemie und Chemische Biologie, Technische Universität Dortmund, Otto-Hahn-Strasse 6, 44227 Dortmund, Germany

**Keywords:** crystal structure, 4-(tri­methyl­germ­yl)benzoic acid, germanium, hydrogen bonding

## Abstract

The title compound, [Ge(CH_3_)_3_(C_7_H_5_O_2_)], was obtained as a by-product in the synthesis of the corresponding aldehyde. Two slightly different mol­ecules are present in the asymmetric unit. In both mol­ecules, the geometry of the aromatic ring plane is distorted by varying intensities. Additionally, the Ge atoms deviate from the mean aromatic ring planes. Whereas the distance of the Ge atom to the ring plane is only 0.101 (4) Å in the first mol­ecule, this distance is increased to 0.210 (4) Å in the second. In the crystal structure, centrosymmetric O—H⋯O hydrogen-bonded dimers are formed. The title compound is isostructural with the Si analogue [Haberecht *et al.* (2004 ▸). *Acta Cryst.* E**60**, o329–0330].

## Abstract 2

During synthesis of the corresponding aldehyde, 4-(tri­methyl­germ­yl)benzoic acid was obtained as a side-product. It crystallizes with two independent mol­ecules in the asymmetric unit which exhibit slightly different geometries. In the crystal structure, centrosymmetric hydrogen-bonded dimers of the mol­ecular pairs are formed.

## Chemical context   

The application of 1,4-di­hydro­pyridines (DHPs) as a pharmaceutical tool represents a novel and promising approach in the therapy of autoimmune diseases, cancer and other illnesses. The effect of drugs containing DHPs is based on the inter­action with the Transforming Growth Factor β (TGFβ). The title compound, [Ge(CH_3_)_3_(C_7_H_5_O_2_)], (I), was obtained as a side-product in the synthesis of the corresponding aldehyde, which can be employed in the synthesis of DHPs (Längle *et al.*, 2015 ▸).
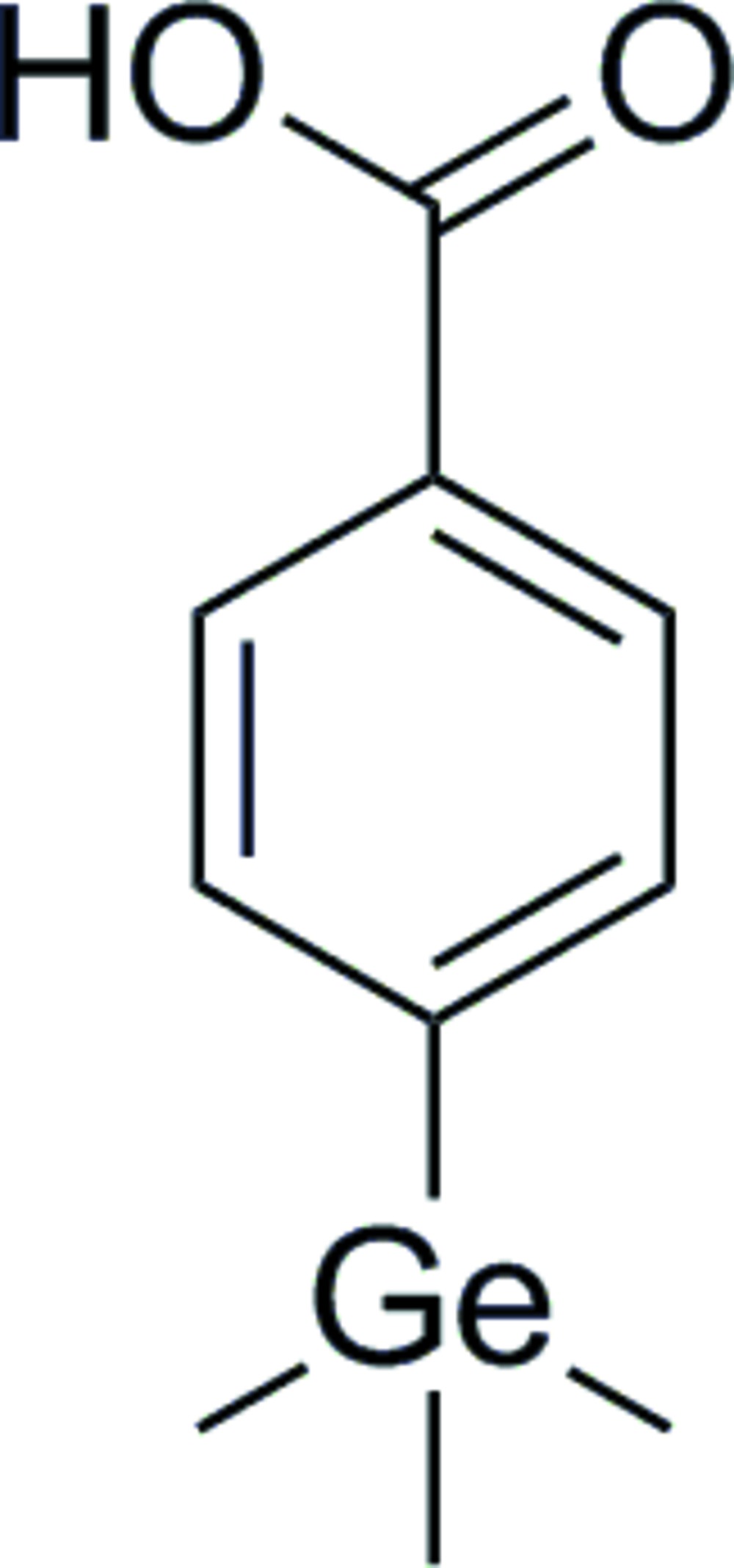


## Structural commentary   

**Figure 1:**
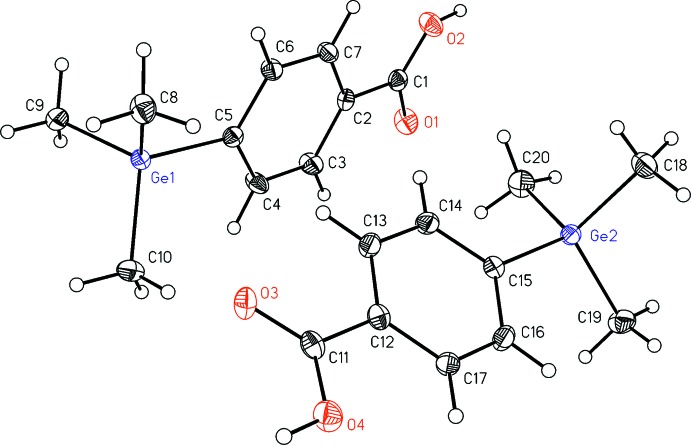
The structures and atom numbering of the two independent mol­ecules in the title compound. Displacement ellipsoids are drawn at the 30% probability level.

The asymmetric unit of (I) contains two mol­ecules (Fig. 1 ▸), which exhibit different deformations of the aromatic plane. This deformation may be caused by the sterically demanding substituents in 1- and 4-positions. In the first mol­ecule, the opposite carbon atoms C2 and C5 deviate from the mean aromatic ring plane by −0.015 (2) Å, which leads to a boat-shaped deformation (Table 1 ▸). The distance of the germanium atom Ge1 to this plane is −0.210 (4) Å. Corresponding to this boat-shaped deformation, the bond lengths of the aromatic ring are not equidistant, but can be divided into three pairs of similar distances: the bonds C5—C4 [1.393 (4) Å] and C5—C6 [1.398 (4) Å] are slightly elongated, C2—C3 [1.383 (4) Å] and C2—C7 [1.384 (4) Å] lie in a medium range, and C3—C4 [1.368 (4) Å] and C6—C7 [1.379 (4) Å] are the shortest. In the second mol­ecule, the aromatic ring exhibits a nearly planar geometry (Table 1 ▸). Similar to the first mol­ecule, the Ge2 atom deviates from the mean aromatic ring plane by 0.101 (4) Å. Additionally, elongated bond lengths at C12 and C15 can be observed [C12—C13 1.385 (4), C12—C17 1.381 (4), C15—C14 1.393 (4), C15—C16 1.398 (4) Å].

All in all, the degree of deformation in the second mol­ecule is smaller compared to the first mol­ecule. This difference may be the reason for the presence of two mol­ecules in the asymmetric unit. The deformations described above may be caused by the sterically demanding substituents attached to the aromatic ring in 1- and 4-positions, or may be traced back to packing effects.

## Supra­molecular features   

**Figure 2:**
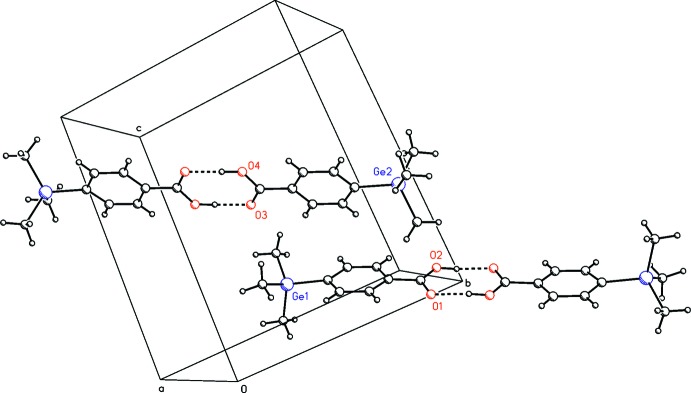
Illustration of the hydrogen-bonded dimers in the unit cell. Hydrogen bonds are represented as dashed lines.

The mol­ecules in the title compound crystallize as centrosymmetric hydrogen-bonded dimers (Fig. 2 ▸, Table 2 ▸). Considering the donor⋯acceptor bond lengths of 2.626 (3) Å [O2—H2⋯O1] and 2.635 (3) Å [O4—H4*A*⋯O3], the strength of the hydrogen bonds can be classified as moderate according to Jeffrey (1997 ▸).

## Database survey   

In the isotypic structure containing silicon instead of germanium, similar distortions can be observed (Haberecht *et al.*, 2004 ▸). In this structure, the asymmetric unit also contains two differently deformed mol­ecules. In the first mol­ecule, a nearly planar geometry of the aromatic ring plane is exhibited. The second mol­ecule shows the same boat-shaped deformation of the aromatic ring as described for the Ge compound. The atoms equal to C12 and C15 deviate by −0.016 (1) Å and −0.017 (1) Å, respectively. The silicon atoms Si1 and Si2 exhibit distances to the aromatic ring plane of 0.088 (3) and −0.219 (2) Å, respectively. A comparison of these distances to those reported for the title compound reveals that the observed distortions occur in similar dimensions for both structures. This points to a comparable steric demand of the tri­methyl­germyl and tri­methyl­silyl moieties.

## Synthesis and crystallization   

To a solution of 1,4-di­bromo­benzene (1.50 g, 6.36 mmol) in Et_2_O (13 ml) was added *n*-BuLi (6.36 mmol, 2.5 *M* in hexa­ne) at 195 K and the mixture stirred at this temperature for 4 h. Then chloro­tri­methyl­germane (1.10 g, 7.00 mmol) was added to the reaction mixture at 195 K, stirred at this temperature for 10 min, followed by stirring over night at room temperature. After addition of H_2_O, the organic phase was separated and the aqueous phase was extracted with Et_2_O three times. The combined organic phases were washed with brine and dried over Na_2_SO_4_. Removal of the solvent under reduced pressure afforded (4-bromo­phen­yl)tri­methyl­germane (1.67 g, 6.12 mmol, 96%) as a colorless liquid. The reaction product was used in following syntheses without further purification.

**Figure 3:**
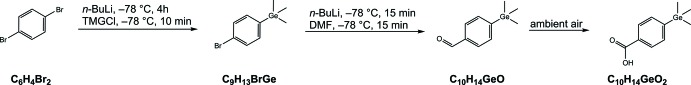
Schematic representation of the synthesis of compound (I).

To a solution of (4-bromo­phen­yl)tri­methyl­germane (1.67 g, 6.12 mmol) in THF (38 ml) was added *n*-BuLi (6.73 mmol, 2.5 *M* in hexa­ne) at 195 K and the mixture was stirred at this temperature for 15 minutes. Then di­methyl­formamide (1.34 g, 18.4 mmol) was added to the reaction mixture at 195 K, and it was allowed to warm to room temperature over night. After addition of a saturated aqueous NH_4_Cl solution, the organic phase was separated and the aqueous phase extracted three times with Et_2_O. The combined organic phases were washed with water and brine and dried over Na_2_SO_4_. Removal of the solvent under reduced pressure and subsequent silica gel chromatography (pentane, penta­ne/Et_2_O = 100:1 → 50:1) afforded 4-(tri­methyl­germ­yl)benzaldehyde, which oxidized at ambient air conditions to give 4-(tri­methyl­germ­yl)benzoic acid, (I), (1.05 g, 4.70 mmol, 77%) as a colorless solid. A schematic representation of the synthetic procedure is shown in Fig. 3 ▸.

## Refinement   

Crystal data, data collection and structure refinement details are summarized in Table 3 ▸. Hydrogen atoms were located from difference Fourier maps. They were refined with ideal­ized positions in a riding model with *U*_iso_(H) = 1.2*U*_eq_(C) and C—H = 0.95 Å for aromatic hydrogen atoms, and with *U*_iso_(H) = 1.5*U*_eq_(C) and C—H = 0.98 Å for methyl hydrogen atoms. All CH_3_ hydrogen atoms were allowed to rotate but not to tip. Hydroxyl hydrogen atoms were located from difference Fourier maps and were refined freely.

## Supplementary Material

Crystal structure: contains datablock(s) I. DOI: 10.1107/S2056989015009160/wm5155sup1.cif

Structure factors: contains datablock(s) I. DOI: 10.1107/S2056989015009160/wm5155Isup2.hkl

Supporting information file. DOI: 10.1107/S2056989015009160/wm5155Isup3.cml

CCDC reference: 1400647

Additional supporting information:  crystallographic information; 3D view; checkCIF report

## Figures and Tables

**Table 1:** Deviation of atoms from the benzene ring least-squares planes ()

Atom	Deviation	Atom	Deviation
C2	0.015(2)	C12	0.004(2)
C3	0.007(2)	C13	0.003(2)
C4	0.008(2)	C14	0.003(2)
C5	0.015(2)	C15	0.007(2)
C6	0.007(2)	C16	0.006(2)
C7	0.007(2)	C17	0.000(4)
Ge1*	0.210(4)	Ge2*	0.101(4)

Note: (*) not used in the least-squares-plane calculation.

**Table 2:** Hydrogen-bond geometry (, )

*D*H*A*	*D*H	H*A*	*D* *A*	*D*H*A*
O2H2O1^i^	0.93(5)	1.71(5)	2.626(3)	170(5)
O4H4*A*O3^ii^	0.93(5)	1.70(5)	2.635(3)	179(4)

Symmetry codes: (i) 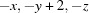; (ii) 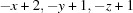.

**Table 3:** Experimental details

Crystal data	
Chemical formula	[Ge(CH_3_)_3_(C_7_H_5_O_2_)]
*M*_r_	238.80
Crystal system, space group	Triclinic, *P* 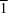
Temperature (K)	173
*a*, *b*, *c* ()	6.3560(4), 12.3927(6), 14.2084(7)
, , ()	96.348(4), 92.846(4), 93.246(4)
*V* (^3^)	1108.76(10)
*Z*	4
Radiation type	Mo *K*
(mm^1^)	2.73
Crystal size (mm)	0.08 0.08 0.02

Data collection	
Diffractometer	Agilent Xcalibur Sapphire3
Absorption correction	Multi-scan (*CrysAlis PRO*; Agilent, 2014 ▸)
*T*_min_, *T*_max_	0.794, 1.000
No. of measured, independent and observed [*I* > 2(*I*)] reflections	15667, 4781, 3261
*R*_int_	0.044
(sin /)_max_ (^1^)	0.639

Refinement	
*R*[*F* ^2^ > 2(*F* ^2^)], *wR*(*F* ^2^), *S*	0.036, 0.083, 1.02
No. of reflections	4781
No. of parameters	249
H-atom treatment	H atoms treated by a mixture of independent and constrained refinement
_max_, _min_ (e ^3^)	0.54, 0.31

Computer programs: *CrysAlis PRO* (Agilent, 2014 ▸), *SHELXS97* (Sheldrick, 2008 ▸), *SHELXL2014* (Sheldrick, 2015 ▸) and *OLEX2* (Dolomanov *et al.*, 2009 ▸).

## supplementary crystallographic information

### Crystal data

**Table:**

[Ge(CH_3_)_3_(C_7_H_5_O_2_)]	*Z* = 4
*M**_r_* = 238.80	*F*(000) = 488
Triclinic, *P*1	*D*_x_ = 1.431 Mg m^−^^3^
*a* = 6.3560 (4) Å	Mo *K*α radiation, λ = 0.71073 Å
*b* = 12.3927 (6) Å	Cell parameters from 4611 reflections
*c* = 14.2084 (7) Å	θ = 2.9–28.4°
α = 96.348 (4)°	µ = 2.73 mm^−^^1^
β = 92.846 (4)°	*T* = 173 K
γ = 93.246 (4)°	Plate, clear colourless
*V* = 1108.76 (10) Å^3^	0.08 × 0.08 × 0.02 mm

### Data collection

**Table:**

Agilent Xcalibur Sapphire3 diffractometer	4781 independent reflections
Radiation source: Enhance (Mo) X-ray Source	3261 reflections with *I* > 2σ(*I*)
Graphite monochromator	*R*_int_ = 0.044
Detector resolution: 16.0560 pixels mm^-1^	θ_max_ = 27.0°, θ_min_ = 2.3°
ω scans	*h* = −8→8
Absorption correction: multi-scan (*CrysAlis PRO*; Agilent, 2014)	*k* = −15→15
*T*_min_ = 0.794, *T*_max_ = 1.000	*l* = −18→18
15667 measured reflections	

### Refinement

**Table:**

Refinement on *F*^2^	Primary atom site location: structure-invariant direct methods
Least-squares matrix: full	Hydrogen site location: mixed
*R*[*F*^2^ > 2σ(*F*^2^)] = 0.036	H atoms treated by a mixture of independent and constrained refinement
*wR*(*F*^2^) = 0.083	*w* = 1/[σ^2^(*F*_o_^2^) + (0.0332*P*)^2^] where *P* = (*F*_o_^2^ + 2*F*_c_^2^)/3
*S* = 1.01	(Δ/σ)_max_ < 0.001
4781 reflections	Δρ_max_ = 0.54 e Å^−^^3^
249 parameters	Δρ_min_ = −0.31 e Å^−^^3^
0 restraints	

### Special details

**Table:**

Geometry. All e.s.d.'s (except the e.s.d. in the dihedral angle between two l.s. planes) are estimated using the full covariance matrix. The cell e.s.d.'s are taken into account individually in the estimation of e.s.d.'s in distances, angles and torsion angles; correlations between e.s.d.'s in cell parameters are only used when they are defined by crystal symmetry. An approximate (isotropic) treatment of cell e.s.d.'s is used for estimating e.s.d.'s involving l.s. planes.

### Fractional atomic coordinates and isotropic or equivalent isotropic displacement parameters (Å^2^)

**Table:**

	*x*	*y*	*z*	*U*_iso_*/*U*_eq_	
Ge1	0.34904 (5)	0.43946 (2)	0.19617 (2)	0.02258 (10)	
O1	0.1858 (3)	0.90478 (18)	−0.01761 (15)	0.0386 (6)	
O2	−0.0693 (4)	0.9178 (2)	0.08546 (16)	0.0369 (6)	
H2	−0.124 (7)	0.976 (4)	0.060 (3)	0.13 (2)*	
C1	0.0846 (5)	0.8712 (3)	0.0511 (2)	0.0268 (7)	
C2	0.1569 (5)	0.7738 (2)	0.0910 (2)	0.0237 (7)	
C3	0.3337 (5)	0.7244 (3)	0.0588 (2)	0.0309 (8)	
H3	0.4140	0.7559	0.0131	0.037*	
C4	0.3942 (5)	0.6307 (3)	0.0920 (2)	0.0306 (8)	
H4	0.5164	0.5985	0.0688	0.037*	
C5	0.2814 (4)	0.5813 (2)	0.1589 (2)	0.0230 (7)	
C6	0.1072 (4)	0.6339 (2)	0.1931 (2)	0.0259 (7)	
H6	0.0291	0.6039	0.2404	0.031*	
C7	0.0460 (5)	0.7283 (2)	0.1598 (2)	0.0262 (7)	
H7	−0.0732	0.7624	0.1843	0.031*	
C8	0.2619 (5)	0.4263 (3)	0.3237 (2)	0.0355 (8)	
H8A	0.3064	0.3576	0.3438	0.053*	
H8B	0.1079	0.4275	0.3244	0.053*	
H8C	0.3273	0.4872	0.3672	0.053*	
C9	0.1964 (5)	0.3288 (3)	0.1066 (2)	0.0324 (8)	
H9A	0.2346	0.3388	0.0421	0.049*	
H9B	0.0442	0.3351	0.1115	0.049*	
H9C	0.2334	0.2565	0.1213	0.049*	
C10	0.6506 (4)	0.4247 (3)	0.1914 (2)	0.0361 (8)	
H10A	0.7241	0.4701	0.2453	0.054*	
H10B	0.6991	0.4480	0.1320	0.054*	
H10C	0.6808	0.3484	0.1945	0.054*	
Ge2	0.64145 (5)	1.05828 (3)	0.30054 (2)	0.02464 (10)	
O3	0.7978 (3)	0.54301 (17)	0.43295 (15)	0.0338 (5)	
O4	1.0933 (4)	0.6355 (2)	0.49737 (16)	0.0376 (6)	
H4A	1.133 (7)	0.573 (4)	0.522 (3)	0.116 (19)*	
C11	0.9143 (5)	0.6285 (3)	0.4488 (2)	0.0267 (7)	
C12	0.8508 (5)	0.7298 (3)	0.4112 (2)	0.0250 (7)	
C13	0.6552 (5)	0.7320 (2)	0.3641 (2)	0.0276 (7)	
H13	0.5620	0.6686	0.3551	0.033*	
C14	0.5962 (5)	0.8266 (2)	0.3304 (2)	0.0275 (7)	
H14	0.4618	0.8271	0.2981	0.033*	
C15	0.7279 (5)	0.9214 (2)	0.3421 (2)	0.0244 (7)	
C16	0.9254 (5)	0.9163 (3)	0.3887 (2)	0.0306 (8)	
H16	1.0205	0.9790	0.3968	0.037*	
C17	0.9849 (5)	0.8226 (2)	0.4229 (2)	0.0278 (7)	
H17	1.1195	0.8215	0.4549	0.033*	
C18	0.4858 (5)	1.1327 (3)	0.4005 (2)	0.0401 (9)	
H18A	0.3884	1.0801	0.4254	0.060*	
H18B	0.4055	1.1890	0.3746	0.060*	
H18C	0.5851	1.1665	0.4517	0.060*	
C19	0.8925 (5)	1.1465 (3)	0.2791 (2)	0.0383 (9)	
H19A	0.9715	1.1690	0.3398	0.057*	
H19B	0.8517	1.2110	0.2503	0.057*	
H19C	0.9813	1.1039	0.2365	0.057*	
C20	0.4583 (5)	1.0264 (3)	0.1866 (2)	0.0404 (9)	
H20A	0.5249	0.9760	0.1408	0.061*	
H20B	0.4346	1.0940	0.1588	0.061*	
H20C	0.3229	0.9932	0.2024	0.061*	

### Atomic displacement parameters (Å^2^)

**Table:**

	*U*^11^	*U*^22^	*U*^33^	*U*^12^	*U*^13^	*U*^23^
Ge1	0.02235 (19)	0.02012 (19)	0.02573 (19)	0.00270 (14)	0.00329 (14)	0.00304 (14)
O1	0.0510 (15)	0.0342 (14)	0.0335 (13)	0.0061 (12)	0.0093 (12)	0.0124 (11)
O2	0.0398 (14)	0.0324 (14)	0.0416 (14)	0.0141 (12)	0.0061 (12)	0.0108 (12)
C1	0.0287 (18)	0.0262 (19)	0.0240 (17)	−0.0001 (14)	−0.0020 (14)	−0.0015 (14)
C2	0.0280 (17)	0.0190 (16)	0.0232 (16)	−0.0010 (13)	−0.0015 (13)	0.0000 (13)
C3	0.0338 (18)	0.033 (2)	0.0291 (18)	0.0054 (15)	0.0141 (15)	0.0104 (15)
C4	0.0319 (18)	0.0303 (19)	0.0329 (18)	0.0105 (15)	0.0142 (15)	0.0078 (15)
C5	0.0235 (16)	0.0242 (17)	0.0206 (16)	−0.0005 (13)	0.0003 (13)	0.0012 (13)
C6	0.0297 (17)	0.0253 (18)	0.0240 (16)	0.0025 (14)	0.0102 (14)	0.0048 (14)
C7	0.0258 (16)	0.0241 (17)	0.0298 (17)	0.0061 (13)	0.0058 (14)	0.0042 (14)
C8	0.045 (2)	0.0308 (19)	0.0326 (19)	0.0042 (16)	0.0076 (16)	0.0084 (15)
C9	0.0279 (18)	0.0293 (19)	0.0386 (19)	−0.0012 (14)	0.0035 (15)	−0.0016 (15)
C10	0.0236 (17)	0.039 (2)	0.046 (2)	0.0056 (15)	0.0030 (15)	0.0063 (17)
Ge2	0.02168 (19)	0.0210 (2)	0.0313 (2)	0.00206 (14)	−0.00119 (14)	0.00386 (15)
O3	0.0441 (14)	0.0212 (12)	0.0352 (13)	0.0003 (11)	−0.0028 (11)	0.0024 (10)
O4	0.0411 (15)	0.0338 (15)	0.0379 (14)	0.0072 (12)	−0.0082 (11)	0.0060 (12)
C11	0.0357 (19)	0.0264 (19)	0.0187 (16)	0.0078 (15)	0.0043 (14)	0.0010 (14)
C12	0.0315 (18)	0.0261 (18)	0.0173 (15)	0.0040 (14)	0.0043 (13)	−0.0011 (13)
C13	0.0284 (17)	0.0234 (17)	0.0302 (18)	−0.0018 (14)	0.0000 (14)	0.0020 (14)
C14	0.0237 (17)	0.0269 (18)	0.0320 (18)	0.0025 (14)	−0.0028 (14)	0.0052 (14)
C15	0.0257 (17)	0.0225 (17)	0.0250 (17)	0.0037 (13)	0.0019 (13)	0.0018 (14)
C16	0.0299 (18)	0.0241 (18)	0.0363 (19)	−0.0045 (14)	−0.0038 (15)	0.0020 (15)
C17	0.0250 (17)	0.0258 (18)	0.0321 (18)	0.0041 (14)	−0.0059 (14)	0.0030 (14)
C18	0.035 (2)	0.040 (2)	0.044 (2)	0.0114 (16)	0.0014 (17)	−0.0034 (17)
C19	0.0310 (19)	0.032 (2)	0.054 (2)	−0.0012 (15)	0.0039 (17)	0.0172 (17)
C20	0.042 (2)	0.040 (2)	0.039 (2)	0.0076 (17)	−0.0100 (17)	0.0072 (17)

### Geometric parameters (Å, º)

**Table:**

Ge1—C5	1.955 (3)	Ge2—C15	1.955 (3)
Ge1—C8	1.942 (3)	Ge2—C18	1.949 (3)
Ge1—C9	1.945 (3)	Ge2—C19	1.938 (3)
Ge1—C10	1.939 (3)	Ge2—C20	1.937 (3)
O1—C1	1.289 (3)	O3—C11	1.250 (4)
O2—H2	0.93 (5)	O4—H4A	0.93 (5)
O2—C1	1.256 (3)	O4—C11	1.295 (4)
C1—C2	1.476 (4)	C11—C12	1.486 (4)
C2—C3	1.383 (4)	C12—C13	1.385 (4)
C2—C7	1.384 (4)	C12—C17	1.381 (4)
C3—H3	0.9500	C13—H13	0.9500
C3—C4	1.368 (4)	C13—C14	1.379 (4)
C4—H4	0.9500	C14—H14	0.9500
C4—C5	1.393 (4)	C14—C15	1.393 (4)
C5—C6	1.398 (4)	C15—C16	1.398 (4)
C6—H6	0.9500	C16—H16	0.9500
C6—C7	1.379 (4)	C16—C17	1.374 (4)
C7—H7	0.9500	C17—H17	0.9500
C8—H8A	0.9800	C18—H18A	0.9800
C8—H8B	0.9800	C18—H18B	0.9800
C8—H8C	0.9800	C18—H18C	0.9800
C9—H9A	0.9800	C19—H19A	0.9800
C9—H9B	0.9800	C19—H19B	0.9800
C9—H9C	0.9800	C19—H19C	0.9800
C10—H10A	0.9800	C20—H20A	0.9800
C10—H10B	0.9800	C20—H20B	0.9800
C10—H10C	0.9800	C20—H20C	0.9800
			
C8—Ge1—C5	109.93 (12)	C18—Ge2—C15	108.31 (13)
C8—Ge1—C9	109.99 (13)	C19—Ge2—C15	108.52 (13)
C9—Ge1—C5	107.50 (13)	C19—Ge2—C18	110.01 (15)
C10—Ge1—C5	109.30 (13)	C20—Ge2—C15	108.88 (13)
C10—Ge1—C8	109.89 (14)	C20—Ge2—C18	109.23 (14)
C10—Ge1—C9	110.19 (13)	C20—Ge2—C19	111.81 (15)
C1—O2—H2	122 (3)	C11—O4—H4A	116 (3)
O1—C1—C2	117.5 (3)	O3—C11—O4	123.6 (3)
O2—C1—O1	123.2 (3)	O3—C11—C12	120.3 (3)
O2—C1—C2	119.3 (3)	O4—C11—C12	116.0 (3)
C3—C2—C1	120.8 (3)	C13—C12—C11	120.0 (3)
C3—C2—C7	118.6 (3)	C17—C12—C11	120.6 (3)
C7—C2—C1	120.6 (3)	C17—C12—C13	119.3 (3)
C2—C3—H3	119.6	C12—C13—H13	120.1
C4—C3—C2	120.8 (3)	C14—C13—C12	119.7 (3)
C4—C3—H3	119.6	C14—C13—H13	120.1
C3—C4—H4	119.1	C13—C14—H14	119.0
C3—C4—C5	121.7 (3)	C13—C14—C15	122.0 (3)
C5—C4—H4	119.1	C15—C14—H14	119.0
C4—C5—Ge1	121.8 (2)	C14—C15—Ge2	122.6 (2)
C4—C5—C6	116.8 (3)	C14—C15—C16	117.0 (3)
C6—C5—Ge1	121.2 (2)	C16—C15—Ge2	120.4 (2)
C5—C6—H6	119.3	C15—C16—H16	119.3
C7—C6—C5	121.5 (3)	C17—C16—C15	121.4 (3)
C7—C6—H6	119.3	C17—C16—H16	119.3
C2—C7—H7	119.8	C12—C17—H17	119.7
C6—C7—C2	120.4 (3)	C16—C17—C12	120.6 (3)
C6—C7—H7	119.8	C16—C17—H17	119.7
Ge1—C8—H8A	109.5	Ge2—C18—H18A	109.5
Ge1—C8—H8B	109.5	Ge2—C18—H18B	109.5
Ge1—C8—H8C	109.5	Ge2—C18—H18C	109.5
H8A—C8—H8B	109.5	H18A—C18—H18B	109.5
H8A—C8—H8C	109.5	H18A—C18—H18C	109.5
H8B—C8—H8C	109.5	H18B—C18—H18C	109.5
Ge1—C9—H9A	109.5	Ge2—C19—H19A	109.5
Ge1—C9—H9B	109.5	Ge2—C19—H19B	109.5
Ge1—C9—H9C	109.5	Ge2—C19—H19C	109.5
H9A—C9—H9B	109.5	H19A—C19—H19B	109.5
H9A—C9—H9C	109.5	H19A—C19—H19C	109.5
H9B—C9—H9C	109.5	H19B—C19—H19C	109.5
Ge1—C10—H10A	109.5	Ge2—C20—H20A	109.5
Ge1—C10—H10B	109.5	Ge2—C20—H20B	109.5
Ge1—C10—H10C	109.5	Ge2—C20—H20C	109.5
H10A—C10—H10B	109.5	H20A—C20—H20B	109.5
H10A—C10—H10C	109.5	H20A—C20—H20C	109.5
H10B—C10—H10C	109.5	H20B—C20—H20C	109.5
			
Ge1—C5—C6—C7	−173.3 (2)	Ge2—C15—C16—C17	176.5 (2)
O1—C1—C2—C3	3.4 (4)	O3—C11—C12—C13	4.5 (4)
O1—C1—C2—C7	−175.0 (3)	O3—C11—C12—C17	−175.6 (3)
O2—C1—C2—C3	−176.0 (3)	O4—C11—C12—C13	−175.5 (3)
O2—C1—C2—C7	5.6 (5)	O4—C11—C12—C17	4.4 (4)
C1—C2—C3—C4	−176.4 (3)	C11—C12—C13—C14	179.3 (3)
C1—C2—C7—C6	176.4 (3)	C11—C12—C17—C16	−179.6 (3)
C2—C3—C4—C5	0.1 (5)	C12—C13—C14—C15	−0.1 (5)
C3—C2—C7—C6	−2.0 (5)	C13—C12—C17—C16	0.3 (4)
C3—C4—C5—Ge1	173.2 (2)	C13—C14—C15—Ge2	−176.8 (2)
C3—C4—C5—C6	−2.1 (5)	C13—C14—C15—C16	1.0 (4)
C4—C5—C6—C7	2.1 (4)	C14—C15—C16—C17	−1.3 (4)
C5—C6—C7—C2	0.0 (5)	C15—C16—C17—C12	0.7 (5)
C7—C2—C3—C4	2.0 (5)	C17—C12—C13—C14	−0.6 (4)

### Hydrogen-bond geometry (Å, º)

**Table:**

*D*—H···*A*	*D*—H	H···*A*	*D*···*A*	*D*—H···*A*
O2—H2···O1^i^	0.93 (5)	1.71 (5)	2.626 (3)	170 (5)
O4—H4*A*···O3^ii^	0.93 (5)	1.70 (5)	2.635 (3)	179 (4)

Symmetry codes: (i) −*x*, −*y*+2, −*z*; (ii) −*x*+2, −*y*+1, −*z*+1.
